# Vulcanite Work

**Published:** 1861-08

**Authors:** 


### VULCANITE WORK.

We have, from time to time, as occasion seemed to require, given our views on the use of vulcanized rubber for dental purposes, and the mode of working it. In regard to its value and efficiency, we have nothing new to offer. It seems to be in regard to permanency, all that we could have anticipated. The facility of working it is much greater now than formerly, the difficulties that were at first to contend with have almost all been removed, so that this style of work is now as easily accomplished as any other.

Almost all who have attempted it have found the same difficulties to contend with ; many have cleared all these away, and now go smoothly on ; others became discouraged, and abandoned it.

We are often written to in regard to the present mode of working vulcanite. There is no very obvious difference between the present method of working, and that of two years ago, except the process of vulcanizing. The other particulars of difference are made up of *little* things—peculiarities of manipulation that are difficult to describe, indeed, must be seen, to be fully understood, and which practice only will enable any one to attain; such for instance, as packing in the proper amount of rubber, for any given case, and warming up to the proper temperature, and closing the flask, etc.

We propose here to give our present method of vulcanizing as it differs somewhat from any we have given heretofore.

We use the three piece copper, gas or alcohol vulcanizer put in water sufficient to cover the piece or pieces to be vulcanized, and close up immediately; be sure that the safety valve and the thermometer are all right. In regard to the safety valve, this may be determined by working it with the fingers; close inspection will sometimes detect an injury of the thermometer, but not always; any injury, however, may be positively determined by bringing up the heat till the steam escapes from the safety valve. If the mercury does not appear in the tube before this time, it is safe to conclude that the thermometer is broken, or at least not reliable; the heat should at once be removed, the steam permitted to escape, and the thermometer repaired, or replaced by a sound one.

The pieces being placed in the heater, and covered by water, the packing being perfect, the top screwed tightly down, apply the heat, and run the thermometer up to 360° to 380° as quickly as possible; if steam escapes from the safety valve before the desired point has been attained, place a weight upon it, sufficient to keep it perfectly closed. Continue the heat at about the point which is fixed upon, from forty-five to sixty minutes. If the thermometer is at 360°, perhaps sixty to sixty-five minutes will be required to properly vulcanize the American Hard Rubber Company's gum. If the heat is brought up to 380°, forty to fifty minutes will be quite long enough to accomplish the work. There is some variation in this gum. Other gums should, of course, be tested, before entering upon practical work with them.

We know of no better vulcanizer than the one referred to above, and we have tried everything that has been in the market. There have been some complaints made about this machine, but we have yet to learn of *anything* that pleased everybody. Several have written to us recently, making complaints in regard to the vulcanizer. One says, "the thermometers break so easily;" certainly, a man's neck will break if not properly used. Another says, "it is almost impossible to keep them tightly packed." There is no difficulty whatever in this, if the packing is kept in good condition. In all respects, these vulcanizers are as efficient and as easily managed as any similar piece of machinery. T.

